# How ethical is your clinical trial?

**DOI:** 10.1111/j.1742-1241.2010.02421.x

**Published:** 2010-08

**Authors:** L Miller, M Folayan, D Allman, B Nkala, L M Kasirye, L R Mingote, G Calazans, R Mburu, F Ntombela, M Ditmore

**Affiliations:** 1 AVAC: Global Advocacy for HIV PreventionNew York, NY, USA; 2 Department of Child Dental Health, Obafemi Awolowo UniversityIle-lfe, Nigeria; 3 Dalla Lana School of Public Health, HIV Social, Behavioural and Epidemiological Studies Unit, University of TorontoToronto, ON, Canada; 4 Perinatal HIV Research UnitSoweto, South Africa; 5 Makerere University Walter Reed Project, Clinic DepartmentKampala, Uganda; 6 Planeta Salud, Institutional DevelopmentBarcelona, Spain; 7 Centro de Referencia e Treinamento DST/Aids da Secretaria de Estado da Saude de Sao Paulo, Unidade de Pesquisa de Vacinas Anti-HIVSao Paulo, Brazil; 8 Kenya AIDS NGO’s Consortium (KANCO), Policy, Development and AdvocacyNairobi, Kenya; 9 Centre for the AIDS Programme of research in South Africa (CAPRISA), Community ProgrammeCongella, South Africa; 10 National Development Research Institutes, Inc, and Public Health SolutionsNew York, NY, USA

## Abstract

Is Institutional Review Board (IRB) approval and a rigorous informed consent process enough? It is our view that this is no longer the case. Conventional research ethics emphasise the importance of weighing the risks and benefits for prospective participants as one of the key determinants of deeming a clinical trial ethical. We support the notion that ethical obligations of research should include considerations not only at the individual level, but also at the community level (1,2).

As stakeholders in the field of biomedical HIV prevention research, we have seen how the HIV/AIDS pandemic has greatly shaped what is viewed as ethical conduct in research. To assess the effectiveness of new HIV prevention technologies, clinical trials must recruit large numbers of healthy, HIV-negative individuals as study participants. Research ethics stipulate that these new HIV prevention strategies be tested for safety and effectiveness in populations who need these interventions and are likely to use them. Therefore, these trials are conducted often as multi-site, international studies, and with high-incidence populations who may be poor or marginalised and consequently may have less access to standard health care services. Frequently, setting up a biomedical HIV prevention clinical trial is a vast undertaking accompanied by the need for investment in time, human resources and infrastructure. As a result of their size, these trials can have impact on the surrounding community – a new clinic may be built, jobs may be created and access to better health interventions may become available. The arrival of a large trial, however, if not conducted in ways that are sensitive to the local environment and locally determined priorities, may create conflict or misunderstanding between researchers and the community from which trial participants will be recruited.

Through the activism in the 1980’s around AIDS treatment trials, the biomedical field saw, in many ways for the first time, how community stakeholders not associated with the scientific field could play a role in setting the research agenda and making important contributions to the clinical trials process (3). Indeed, communities can play active roles in determining research priorities, and providing important input into trial implementation and monitoring (4). Recently, the need to recognise and standardise the implementation of community participation within the clinical trials process was highlighted through controversies that erupted between researchers and local voices around testing tenofovir disoproxil fumarate (TDF) for the prevention of HIV infection in pre-exposure prophylaxis (PrEP) studies in 2004 and 2005. In Cameroon, the Family Health International PrEP trial was halted by the Minister of Public Health, and in Cambodia, the proposed PrEP trial funded by the US National Institutes of Health and the Bill and Melinda Gates Foundation was never implemented by order of the Cambodian Prime Minister. In Thailand, the US Centers for Disease Control and Prevention PrEP study saw similar differences emerge between community stakeholders and researchers, although the trial was not stopped and is still ongoing.

Analyses of these trials (5–8) indicate that poor communication and a lack of mutual understanding between trial communities and research teams prevented the kinds of positive collaborations that would have been required for such biomedical HIV prevention trials to continue. In the above examples, there was a considerable disconnect between the intentions of researchers and what both local and global community stakeholders felt was happening. Review of these controversies shows clearly that effective communication and building a valid negotiation process are key elements of good community engagement. Such breakdown in communication and understanding which led to the loss of multiple opportunities need not happen.

The PrEP trials in Cameroon, Thailand and Cambodia are case studies of clinical trials reviewed and approved by multiple ethics committees, yet subsequently found unacceptable by some community stakeholders because of differing opinions of ethical requirements for the conduct of research in their communities. Lessons from these trials suggest it can no longer be assumed that all proposed research that requires broad recruitment from communities should be implemented as determined by IRBs and researchers alone. Rather, communities, together with researchers and IRBs, have an important role in determining whether a particular study is appropriate for a particular location and time.

Researchers conducting biomedical HIV prevention research do have an ethical obligation to engage communities. Guidance point two of the UNAIDS/WHO *Ethical Considerations in Biomedical HIV Prevention Trials* (9) clearly states that researchers should consult with communities in a participatory process to ensure ethical and scientific quality of proposed research. *The International Ethical Guidelines for Biomedical Research Involving Human Subjects* by the Council for International Organizations of Medical Sciences (CIOMS) (10) state that research should be responsive to the health needs of communities; consulting with communities on the research process can help ensure that this ethical responsibility is met. Our belief is that these discussions can and should happen early in the process, and in a manner that respects local culture and local priorities. Projects that are more likely to be successful are those that allow researchers and community stakeholders to come together to share ideas on research priorities and how best to conduct trials. A community that is respected and treated as a partner rather than as a supplier of ‘research subjects’ is more likely to be supportive of a proposed clinical trial. Although the process of engaging with communities can never guarantee collaboration free of controversy or free of considerable differences of opinion (11), productive dialogues with community stakeholders can translate into benefits for the research and for communities. Discussions with community stakeholders can help refine study procedures to the local context. This in turn may maximise results (12–15) and lead to more effective recruitment and enrolment strategies, better retention rates and stronger adherence to the study product.

Community engagement is increasingly being recognised as an essential component of ethical conduct of biomedical HIV prevention trials. *Good Participatory Practice Guidelines (GPP) in Biomedical HIV Prevention Trials* (16) is the first set of global guidelines to outline detailed steps to ensure community engagement within the context of biomedical HIV prevention trials. The GPP guidelines are designed for trial sponsors and implementers and identify *core principles* for the foundation of relationships between trial entities and community stakeholders. Examples of such principles are respect, research literacy, ethical and scientific integrity and transparency. The *minimum elements of good community practice* explain how specific activities and actions can facilitate appropriate community engagement at each stage of the trial life-cycle. The GPP guidelines are broad enough to allow for variation in trial sites across the globe, but specific enough to provide a suitable framework to facilitate successful adoption of key activities.

A unique aspect of the GPP guidelines is that they can be utilised as a tool to assess effective collaborative processes by community stakeholders, researchers and trials sponsors. With a potentially powerful tool at the disposal of community stakeholders, it is expected that monitoring of collaborative processes within biomedical HIV prevention trials, as well as other forms of research, will become more rigorous in time.

Institutional Review Board approval of clinical trials is an essential ethical component of the research process. A thorough informed consent process is also essential. However, ethics is a field that evolves and changes over time. In the field of biomedical HIV prevention trials, there has been a shift in the expectations of stakeholders with respect to what is perceived as ethical conduct of research. Support for community engagement as an ethical component of the research process can also be seen outside the field of biomedical HIV prevention research. Increasingly, guidance documents (17,18) are recognising involvement of community stakeholders as an ethical obligation of researchers. At the same time, there are examples of communities in various settings organising themselves to act as self-determining gatekeepers of ethical research – choosing what research they are willing to allow and support within their locales (19,20).

Given these developments, we believe that researchers should embrace this new direction as one possible avenue to enhance the successes of their trials and improve the ethical conduct of their research. They will not be alone. There is existing evidence of the importance and feasibility of such community engagement in the broader health field (21–25). In recent years, funding opportunities for community based and community based participatory research have been added to the portfolios of, among others, the US National Institutes of Health and the Canadian Institutes of Health Research. These developments reinforce the need for institutions to be prepared for these changes and encourage community engagement in the research process. IRB approval can include a wider ethical perspective if relevant clinical trial protocols are required to describe their community engagement plans, and these plans are reviewed for their merit as a criterion in ethical approval.

Engaging in an iterative and collaborative process with community stakeholders is an investment. Discussing research priorities and determining best strategies requires time, effort and financial support. Sponsors and institutions should be prepared to fund activities that can improve trial conduct and lay the foundation for positive collaborations in the future. In genuinely working together with communities, while attending to the best participatory practices possible, researchers, sponsoring institutions and community stakeholders will see more successful clinical trial implementation, more effective controversy mitigation and a greater chance of bringing the field closer to discovering new and vital public health interventions.
